# Lumbar Puncture in the prone position for Low Birth Weight Neonates

**DOI:** 10.1186/s12887-021-03071-7

**Published:** 2022-01-03

**Authors:** Wanxu Guo, Di Ma, Min Qian, Xiaoqi Zhao, Jinpu Zhang, Junjiao Liu, Di Chi, Fengmin Mao, Yunfeng Zhang

**Affiliations:** 1 Department of Neonatology, the Second Hospital of Jilin University, Changchun, 130022 China; 2 Children’s Disease Diagnosis and Treatment Center, the Second Hospital of Jilin University, 218 Ziqiang Street, Changchun, 130022 Jilin Province China

**Keywords:** Lumbar puncture, lateral decubitus position, prone position, success rate, NIAPAS

## Abstract

**Background:**

Lumbar puncture in the lateral decubitus position will make the neonates uncomfortable and is likely to cause position change and unstable vital signs, and the application of sedative drugs will cause adverse effects. This study explored a novel method for lumbar puncture in the prone position for low weight neonates.

**Methods:**

The neonates were randomly assigned into the standard position group receiving lumbar puncture in the lateral decubitus position; and the improved position group receiving lumbar puncture in the prone position. The success rate of first time attempts and the overall success rate of lumbar puncture, incidence of adverse effects, NIAPAS scores were collected and compared between these two groups. The difference in success rate and adverse effects incidence rate was analysed through Chi-square. Student’s t-test was used for the test of NIAPAS rating.

**Results:**

The improved position group had a higher success rate of first attempt and overall success rate, significantly lower incidence of adverse effect and lower NIAPAS scores than those of the standard position group (*P*<0.05).

**Conclusion:**

This lumbar puncture in the prone position is safer, more effective, and more comfortable for preterm neonates and those with low birth weight. Thus, this method is worth of further promotion.

**Trial registration:**

Registration number, ChiCTR2100049923; Date of Registration, August 11, 2021; Retrospectively registered.

## Background

Lumbar puncture is a necessary clinical test for the diagnosis of neurological infection, intracranial hemorrhage and other diseases, as well as, the necessary means of intrathecal injection treatment etc [1]. Currently, the available international standards for lumbar puncture are in the lateral decubitus position, however, neonates have their own unique anatomical and physiological characteristics, lumbar puncture in the lateral decubitus position has the following disadvantages for neonates: 1) When the neonates are held in a modified lateral recumbent position, the neonates cannot cooperate to maintain the position, which will result in struggling and needing the help of an assistant to fix the position [2], thus, it is inevitable to have a change in position caused by struggling [2]; 2) Passive flexion position makes the sick infant uncomfortable, causes position change, and unstable vital signs, moreover, the stress caused by the discomfort affects the long-term prognosis; 3) Application of sedative drugs, such as phenobarbital, to prevent the position change may cause adverse effects. The most frequent adverse effects caused by phenobarbital in neonates include central nervous system depression and respiratory depression which may need additional support [3–5]. A research conducted to explore the effects of different positions (prone, supine, right lateral and left lateral) that preterm infants are placed in during Nasal Continuous Positive Airway Pressure therapy on their vital signs (heart rate, respiration, oxygen saturation) shows that the prone position provides the highest comfort level [6]. Grunau et al.’ research reports that the prone position promotes deep sleep in preterm neonates when they are undisturbed [7]. Moreover, the prone position has pain reducing, comforting and stress-relieving effects in painful procedures [8]. Therefore, we hypothesized that the lumbar puncture in the prone position for the low birth weight neonates would be a better option than that in the lateral decubitus position. In our present study, we compared the success rate of first attempt and overall success rate, incidence of adverse effect and NIAPAS scores for lumbar puncture between the improved group (in the prone position) and the standard group (in the lateral decubitus position) to provide evidence for the promotion of lumbar puncture for low birth weight neonates in the prone position.

## Methods

Aim: To compare the new method with the standard method for effectiveness

Design: The protocol was approved by the Institutional Review Board of Second Hospital of Jilin University and the informed consent was obtained from the parents of the neonates regarding lumber puncture (LP). 171 sick neonates were randomly divided into standard position group and improved position group. A random number generated by a computer was contained in an opaque envelope, each envelope was designated for one participant. Based on the random number, we assigned the odd random number into standard position group, even number into the improved position group. Two doctors were in charge of enrolling and assigning the participants. The other doctors and nurses who were in charge of data collection were blinded from the enrolling and assigning process. The standard position group totalled 89 cases and the improved position group totalled 82. When each neonate was being operated on, a physician and a nurse were assigned to evaluate the pain and presented a pain score by recording the vital signs and facial expression, body movement etc. During the operation process, we implemented the concept of comforting care, that is, to soothe the baby before operation by giving non-nutritive sucking, oral glucose or milk when necessary. Improved position group were then given the procedure in the prone position.

Setting: The lumbar puncture in the lateral decubitus position for neonates has several disadvantages. In the present study, we compared the two positions for lumbar puncture, the lateral decubitus position and the prone position, to explore whether the prone position is feasible and better than the lateral decubitus position. The neonates who were admitted to the Second Hospital of Jilin University between January 2019 to June 2020, whose birth weights were lower than 2500g, and who satisfied the criteria for lumbar puncture indications, were enrolled into the study. Those neonates with lumbar puncture contraindications or birth weight greater than 2500g were excluded from the study.

Sample size calculation: We used the formula to calculate the sample size [9], n=2δ^2^/(μ2-μ1)^2^×f(α, β), Where, n is the sample size for each group; μ1 and μ2 is the expectation of NIAPAS for the improved and standard group, respectively; δ is allowable error, α=0.05, β=0.1. Based on the reference, μ2=7.74, μ1=6.47, δ=2.27, f(0.05, 0.1)=10.5, then n=[2*2.27^2^/(7.74-6.47)^2^]*10.5=67. Considering the possibility of neonate dropout and neonate's compliance, we expanded the sample size to 80 cases in each group. In real practice, we included 171 cases in our study.

## Data Collection

**The operating method for the neonates in the standard position group**:

1. The nurse prepared the lumbar puncture bag for neonates, piezometer tube, sterile gloves, iodophor, adhesive tape etc;
2. The sick neonate was placed in the lateral decubitus position, the assistant helped to fix the sick neonate’s shoulder and buttock with maximal vertebrae flexion;
3. The operator donned a mask, surgical cap and gloves, performed routine disinfection of the puncture site, and put a sterile surgeon drape;
4. The operator used a 24 gauge needle (Closed IV Catheter System) for puncture, marked the entry point on the interspinous space of lumbar 4-5 and the vertebrae midline, perpendicularly inserted the needle slowly, stopped after feeling a breakthrough, observed if cerebrospinal fluid flowed out;
5. The operator collected the cerebrospinal fluid sample, pulled out the needle, pressed the puncture point for 2 minutes, observed if there was any additional fluid outflow, disinfected the puncture area and placed sterile surgical dressing;
6. The punctured baby lied flat without pillow for 4-6 hours [1], the assistant observed the sick neonate’s condition throughout the procedure, recording heart rate, breathing, oxygen saturation, and the neonate’s facial expression and body movement.

**The operating method for the improved position group**:

1. The sick neonate was placed in the prone position with the limbs flexed, unlimited straightening, a butterfly pillow can be placed under the body, all were oriented mainly to the comfort of the neonate. The head of the neonate was tilted to one side to avoid the mouth and nose being pressed, and for the operator to observe the neonate’s condition easily;
2. The operator first stood beside the neonate to soothe (providing a pacifier, milk or 5% glucose);
3. The operator and the nurse ensured that the neonate was comfortable then determined the entry point which was chosen at the intersection between the highest points of the bilateral iliac crests and the lumbar spine midline;
4. The operator disinfected the puncture area and placed surgeon drape, inserted the needle (24 gauge Closed IV Catheter System) perpendicularly to the back direction about 1.0 cm at the puncture site, stopped after feeling a breakthrough, observed if there was cerebrospinal fluid flowing out into the transparent tube;
5. The operator collected the sample for testing, pulled out the needle, pressed the puncture point for 2 minutes, observed if there was any additional fluid outflow from puncture point, disinfected puncture area and placed sterile surgical dressing;
6. The sick neonate lied flat with no pillow for 4-6 hours. The assistant observed the neonate’s state throughout the process, recorded heart rate, breathing, oxygen saturation, and the neonate’s facial expression and body movement.

The information collected from both the standard position group and the improved position group includes: gestational age, birth weight, ratio of boys to girls, postnatal days at the time of the operation. In the operation process, the vital signs and reactions of the sick neonates were recorded. After completion of the operation, these information were collected, success or failure, the number of insertion attempts, any complications (infection, bleeding, spinal nerve damage, epidermoidoma in the spinal canal, apnea, and bradycardia), and pain scale which was assessed by the Neonatal Intensive Acute Pain Assessment Scale (NIAPAS), which includes the following items: facial expression, alertness, crying and screaming, muscle tension, response to operation, heart rate, breathing and blood oxygen saturation and gestational age.

## Statistical analyses

SPSS 21.0 and R 3.6.1 statistical software were used to perform statistical analyses. The difference in success rate and adverse effects incidence rate was analysed through Chi-square. Student’s t-test was used for the test of NIAPAS rating after we tested for the normal distribution and homogeneity of variance of the data. The effects of possible confounders, gestational age, birthweight, indications for LP, ventilation, besides the method of puncture, on the NIAPAS were explored by multiple regression analysis. In addition, the effects of gestational age, birthweight, indications for LP, ventilation on the number of attempts, number of failures were analysed using a Poisson or a negative binomial distribution regression model. Differences were considered to be statistically significant when p value was less than 0.05.

## Results

The sick neonates, who were admitted to our Department from January 2019 to June 2020, and met the criteria for lumbar puncture, totalled 171 cases. Of the 171 sick neonates, 99 were baby boys (57.9%), and 72 were baby girls (42.1%). The mean gestational age at birth was 225.0±23.4days and the mean birth weight was 1411.04±431.5g for the standard position group; the mean gestational age at birth was 218.83±19.4days and the mean birth weight was 1460.67±404.3g for the improved position group. The mean postnatal age was 8 days when lumbar puncture was performed. There was no significant difference in gestational age, birth weight, proportion of boy to girl and proportion of indications for lumbar puncture between the two groups of sick neonates (Table 1).

**Table 1:** Basic Information of sick neonates in the standard position group and improved position group

Parameters	Items	Improved Position Group(n=82)	Standard Position Group (n=89)	χ2/t	*P*
**Sex**	Boy Girl	**47(27.49%)** **35(20.47%)**	**52(30.41%)** **37(21.64%)**	**0.022**	**0.883**
**Mean weight (g)**		**1460.67±404.3**	**1411.04±431.5**	**0.774**	**0.440**
**Mean gestational age (days)**		**218.83±19.4**	**225.00±23.4**	**-2.041**	**0.064**
**Indications for lumbar puncture**	Sepsis	**67(39.2%)**	**70(40.9%)**	**1.580**	**0.442**
	Septicemia	**8(4.7%)**	**7(4.1%)**		
	Purulent meningitis	**1(0.6%)**	**3(1.8%)**		
	Cerebrospinal fluid drainage	**5(2.9%)**	**7(4.1%)**		
	Newborns of mothers with syphilis	**1(0.6%)**	**2(1.2%)**		

There was significant difference in the success rate of first time attempts on the puncture and overall success rate, changes in vital signs, neonatal pain assessment scores. The success rate of the standard position group was 64% (57/89), and the improved position group was 85.3% (70/82) for first time attempts. The overall success rate of the standard position group was 89.9% (80/89) and the improved position group was 97.6% (80/82) (Table 2). For the incidence of adverse effects, the improved position group was significantly lower than that of the standard position group; the improved position group had a total occurrence of 3 cases for adverse effect while the standard position group had 24. The adverse effect for the improved position group was less bleeding when the puncture needle was removed and brief pressure applied to the puncture point, bleeding was eased after pressing again; whereas the major adverse effects of the standard position group were local hemorrhage, bradycardia, unstable transcutaneous oxygen saturation etc. In regards to the NIAPAS score, the standard position group had significantly higher NIAPAS scores than the improved position group, especially the scores on the sub-items of facial expression, crying and screaming, muscle tension, and transcutaneous oxygen saturation.

**Table 2:** Lumbar puncture results for the standard position group and improved position group

Parameters	Standard Position Group (n=89)	Improved Position Group (n=82)	*P* value
No. of Operators	3	2	
Success rate of first time attempt (%)	57(64%)	70(85.3%)	0.001
Overall success rate of two times attempts	80(89.9%)	80(97.6%)	0.041
NIAPAS rating	10.18±4.2	4.72±1.5	0.000
Adverse event incidence (%)	24	3	0.000

Through multiple collinearity diagnosis, the VIF value for gestational age, birthweight, indications for LP, ventilation, is 4.310, 3.775, 1.111, and 1.632, respectively, all less than 10, it is presumed that the data fit the hypothesis of multivariate linear regression analysis, and multicollinearity is absent, it is feasible to perform the operation for multivariate linear regression analysis.

Ventilation support and indications of lumbar puncture fall into multivariate unordered categorical variable, so, ventilation support and indications of lumbar puncture were treated as dummy variable. The results show that gestational age, birth weight can produce effects on NIAPAS (P≤0.001), the remaining factors cannot produce effects on NIAPAS (P>0.05) (Table 3). However, as shown in Table 1, there was no significant difference in gestational age and body weight between these two groups.

**Table 3:** Effects of Confounding factors on NIAPAS using multivariates linear regression analysis

Parameter	Non-standardized Coefficient			Standardized Coffefficient	
	B	Standard error	Beta	T	***P***
Constant	-9.425	5.922		-1.592	.113
Gestational age(Day)	-.005	.002	-.515	-3.500	.001
Birth weight(g)	.113	.031	.585	3.625	.000
Noninvasive ventilation	.647	1.123	.065	.575	.566
Invasive ventilation	1.511	1.721	.109	.878	.381
Sepsis	-1.490	1.265	-.099	-1.178	.241
Purulent meningitis	-.583	2.209	.021	-.264	.792
Cerebrospinal fluid drainage	.071	1.421	.004	.050	.960
Neonate’s mother infected with syphilis	-2.406	2.626	-.074	-.916	.361

The data of number of attempts and number of failure were tested for Kolmogorov-Smirnov, respectively, and results showed that the “number of failure” fitted Poisson distribution, λ=0.32 (P=1.000>0.05), which can be analyzed by Poisson regression analysis; “number of attempts” did not fit Poisson distribution (P<0.001), should be analyzed by negative binomial distribution regression model.

The results of Poisson regression analysis suggest that indication for LP is able to produce effects on number of failure (P<0.001) while other confounders can not (Table 4). The results of negative binomial regression analysis suggest that indication of LP is able to produce effects on number of attempts (P=0.0213<0.05) while other confounders can not (Table 5).

**Table 4:** Poisson regression analysis for number of failures

	Estimate	Std.Error	Z value	Pr(>|z|)
(Intercept)	-2.033	3.5813	-0.721	0.570
Gestational age	-0.003	0.0165	-0.143	0.860
Birthweight	0.001	0.0008	1.319	0.168
Ventilation	0.244	0.3968	0.739	0.539
Indications for LP	0.549	0.1536	2.905	0.000*

**Table 5:** Negative binomial distribution regression analysis for number of attempts

	Estimate	Std.Error	Z value	Pr(>|z|)
(Intercept)	-0.157	0.5730	-0.273	0.785
Gestational age	-8.123e-6	0.0001	-0.073	0.942
Birthweight	0.000	0.0002	1.142	0.253
Ventilation	0.106	0.1538	0.689	0.491
Indications for LP	0.145	0.0630	2.303	0.021*

## Discussion

Our findings show that the lumbar puncture for neonates in the prone position has more advantages than those in the lateral decubitus position. These include a lower rate and fewer of adverse effects, a higher success rate of first attempts, and lower NIAPAS scores.

Although preterm neonates have the nociceptive circuitry required to perceive pain, their sensory systems are functionally immature. An imbalance of excitatory versus inhibitory processes leads to increased nociceptive signaling in the central nervous system [10], which may explain the reason that gestational age and body weight could produce effects on NIAPAS. However, there was no significant difference in gestational age and body weight between these two groups. Therefore, we presume that the method of puncture is the major factor for NIAPAS.

The language ability of neonates is not well developed, so they can not communicate with their parents, which results in poor cooperation. Our results indicate that indications of LP could produce effects on number of attempts and number of failures. We analyzed the possible reason being that the different indication of LP causes different level of pain to neonates, resulting in different cooperation which could affect the number of failures and number of attempts. The mechanisms underlying needs further exploration. There was no significant difference in the distribution of indications of LP between these two groups, thus we believe that the major factor for number of puncture and number of failures is the puncture method.

Apart from the advantages, we also considered the feasibility of the lumbar puncture for neonates in the prone position. When we chose the prone position for the sick neonates and for the purpose of reducing related adverse effects, we also considered the potential risk, whether the puncture point could be palpated if it was safe; whether the cerebrospinal fluid could flow out smoothly; whether the related adverse effects would be increased. To address the questions, we studied the relevant literature and analysed the characteristics of the neonates. Low birth weight neonates have less subcutaneous fat, the interspinous distance can be palpated, and the width of the interspinous distance can meet the needs of puncture. Selim Öncel et al. have done a comparison of different puncture positions for 51 neonates or infants of postnatal ages 1-83 days [11]. According to their findings, even though the interspinous space is the narrowest when lying and stretching the trunk, the width of the space reaches 3.18 mm, compared with the width of 3.445 mm when in a flexion position, the difference is small and will not affect the puncture operation.

The location of the entry point is safe. In comparison with the traditional puncture position, the prone position has little effect on the deflection of the Tuffier’s line (the line between the top of the iliac crests and the lumbar spine midline). The studies carried out by Schoor et al. on 39 neonates found that half of the neonates in the prone position, the Tuffier’s line corresponds to lumbar 4/lumbar 5 interspinous space (20/39), half of them corresponds to the inferior surface of lumbar 4 [12], therefore it is safe to perform puncture.

The normal cerebrospinal fluid pressure of neonates is 80-110 mmH20, that is 8.0-11.0cm H20, the length of the 24 gauge needle we used is about 3.7cm, thus, the cerebrospinal fluid can flow from bottom to top under the normal cerebrospinal fluid pressure.

The prone position does not increase the risk of complications. The common complications of lumbar puncture for the neonates includes: infection, bleeding, spinal nerve damage, apnea and bradycardia. As long as the surgeons follow the aseptic norms and principles, the above mentioned complications will be reduced or avoided.

Usage of the 24 gauge Closed IV Catheter System needle for puncture is possible. With the continual improvement of treatment for neonates, the increase in neonates, in particular the preterm neonates, and low birth weight neonates, there is an increased need for lumbar puncture and cerebrospinal fluid tests. China does not have special lumbar puncture needle for neonates, the minimum traditional lumbar puncture needle is 22 gauge, and its long needle stem affects the entry direction; its softness prevents exertion; moreover, its bluntness makes it difficult to feel the breakthrough during puncture. Therefore, the application of 24 gauge Closed IV Catheter System needle for neonates puncture has gained acceptance [13].

Compared with traditional position for lumbar puncture, the prone position has the following advantages:

Firstly, soothing can reduce the impact of pain stimulation on preterm neonates and produce the benefits of eliminating sick neonate’s anxiety, irritability, and tension etc, and is a simple and economical pain relief method. The common soothing methods include non-nutritional sucking, kangaroo style care, breastfeeding or formula feeding, oral glucose solution or sucrose water etc. These methods make the sick neonates feel comfortable, psychologically satisfied, reduce pain irritation which are beneficial to the smooth completion of the operation, and are conducive to the long-term growth and development of the sick neonates [14].

Secondly, the prone position will help the sick neonate maintain a natural, comfortable position with a high degree of cooperation without the need for sedative drugs, therefore, the adverse effects caused by these drugs can be avoided and the prone position does not need an assistant to help fix the neonate’s position. In contrast, the sick neonate is uncomfortable with the traditional lateral decubitus position, the involuntary movement of the body leads to puncture failure. To maintain position, the assistant often has to exert pressure which further increases the discomfort; or sedative drugs has to be applied, which will produce related adverse effects or puncture failure; this discomfort leads to unstable life vital signs such as transcutaneous oxygen saturation etc, leading more easily to the occurrence of adverse outcomes.

Based on our experiences and study, the prone position for neonate puncture will improve the success rate of first time attempts to perform the puncture, reduce the number of puncture attempts, and decrease the occurrence of adverse events.

## Limitation

Due to the position reason, the method currently cannot be used in cerebrospinal fluid pressure measurement. In addition, the prone position is unsuitable to neonates or neonates with heavy body weight.

## Conclusion

This lumbar puncture in the prone position is safer, more effective, and more comfortable for preterm neonates and those with low birth weight. Therefore, this method is worth of further promotion.

## Data Availability

The datasets used and/or analysed during the current study are available from the corresponding author on reasonable request.
